# Dichloridobis[5-nitro-1-trimethyl­silyl­methyl-1*H*-benzimidazole-κ*N* ^3^]cobalt(II) *N*,*N*-dimethyl­formamide solvate

**DOI:** 10.1107/S1600536810003922

**Published:** 2010-02-06

**Authors:** Mehmet Akkurt, Şerife Pınar Yalçın, Nihat Şireci, Hasan Küçükbay, M. Nawaz Tahir

**Affiliations:** a Department of Physics, Faculty of Arts and Sciences, Erciyes University, 38039 Kayseri, Turkey; b Department of Physics, Faculty of Arts and Sciences, Harran University, 63300 Şanlıurfa, Turkey; c Department of Chemistry, Faculty of Arts and Sciences, Ínönü University, 44280 Malatya, Turkey; d Department of Physics, University of Sargodha, Sargodha, Pakistan

## Abstract

The title compound, [CoCl_2_(C_11_H_15_N_3_O_2_Si)_2_]·C_3_H_7_NO, was synthesized from 5-nitro-1-trimethyl­silylmethyl-1*H*-benzimid­azole and cobalt(II) chloride in dimethyl­formamide. The Co^II^ atom is coordinated in a distorted tetra­hedral environment by two Cl atoms and two N atoms. In the crystal structure, there are a number of C—H⋯Cl and C—H⋯O hydrogen-bonding inter­actions between symmetry-related mol­ecules.

## Related literature

For the structures and properties of benzimidazole compounds and their metal complexes, see: Akkurt *et al.* (2005 ▶); Castro *et al.* (2002 ▶); Küçükbay *et al.* (1996 ▶, 2004 ▶, 2009 ▶); Liu *et al.* (2004 ▶); Lukevics *et al.* (2001 ▶); Pınar *et al.* (2006 ▶); Pan & Xu (2004 ▶); Türktekin *et al.* (2004 ▶); Tavman *et al.* (2005 ▶); Özdemir *et al.* (2005 ▶); Çetinkaya *et al.* (1996 ▶).
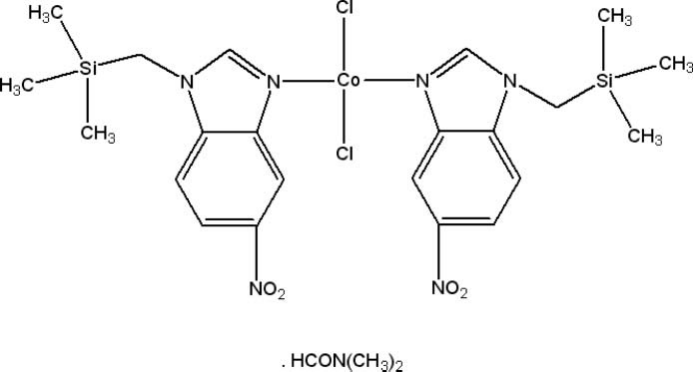


## Experimental

### 

#### Crystal data

[CoCl_2_(C_11_H_15_N_3_O_2_Si)_2_]·C_3_H_7_NO
*M* *_r_* = 701.63
Triclinic, 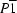

*a* = 9.8982 (4) Å
*b* = 11.6936 (5) Å
*c* = 15.9293 (6) Å
α = 106.041 (2)°
β = 107.408 (2)°
γ = 99.040 (3)°
*V* = 1631.97 (12) Å^3^
*Z* = 2
Mo *K*α radiation
μ = 0.81 mm^−1^
*T* = 100 K
0.20 × 0.12 × 0.08 mm

#### Data collection

Bruker APEXII QUAZAR diffractometer
Absorption correction: multi-scan (*SADABS*; Bruker, 2008 ▶) *T*_min_ = 0.890, *T*_max_ = 0.937
30895 measured reflections
8851 independent reflections
5342 reflections with *I* > 2σ(*I*)
*R*_int_ = 0.059

#### Refinement

*R*[*F* ^2^ > 2σ(*F* ^2^)] = 0.053
*wR*(*F* ^2^) = 0.123
*S* = 1.02
8851 reflections
387 parameters
H-atom parameters constrained
Δρ_max_ = 0.90 e Å^−3^
Δρ_min_ = −0.48 e Å^−3^

### 

Data collection: *APEX2* (Bruker, 2009 ▶); cell refinement: *SAINT* (Bruker, 2009 ▶); data reduction: *SAINT*; program(s) used to solve structure: *SIR97* (Altomare *et al.*, 1999 ▶); program(s) used to refine structure: *SHELXL97* (Sheldrick, 2008 ▶); molecular graphics: *ORTEP-3 for Windows* (Farrugia, 1997 ▶) and *PLATON* (Spek, 2009 ▶); software used to prepare material for publication: *WinGX* (Farrugia, 1999 ▶) and *PLATON*.

## Supplementary Material

Crystal structure: contains datablocks global, I. DOI: 10.1107/S1600536810003922/bt5186sup1.cif

Structure factors: contains datablocks I. DOI: 10.1107/S1600536810003922/bt5186Isup2.hkl

Additional supplementary materials:  crystallographic information; 3D view; checkCIF report

## Figures and Tables

**Table 1:** Hydrogen-bond geometry (Å, °)

*D*—H⋯*A*	*D*—H	H⋯*A*	*D*⋯*A*	*D*—H⋯*A*
C5—H5⋯Cl1^i^	0.95	2.79	3.564 (3)	140
C7—H7⋯O5^ii^	0.95	2.32	3.132 (4)	143
C8—H8*B*⋯O5^ii^	0.99	2.54	3.406 (4)	145
C19—H19*A*⋯Cl2^iii^	0.99	2.67	3.659 (3)	175
C19—H19*B*⋯O4^i^	0.99	2.38	3.182 (4)	137
C22—H22*C*⋯Cl1^iv^	0.98	2.82	3.695 (3)	149
C24—H24*A*⋯O5	0.98	2.42	2.793 (5)	102

Symmetry codes: (i) 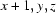; (ii) 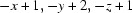; (iii) 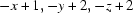; (iv) 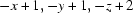.

**Fig. 1.**
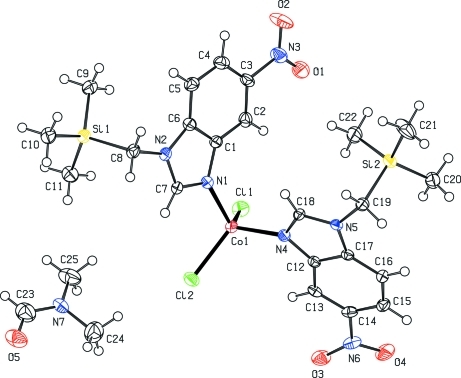
The title molecule showing the atom-labelling scheme. The probability level for the anisotropic displacement parameters is at 50%.

**Fig. 2.**
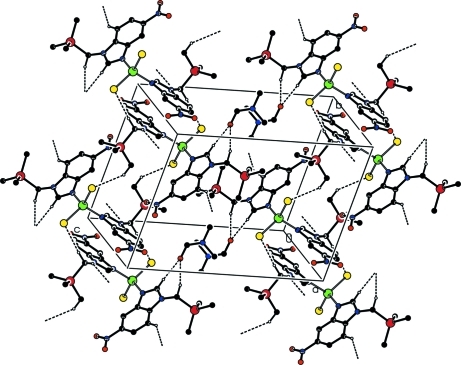
View of the packing diagram of the title compound in the unit cell. Hydrogen bonds are indicated as dashed lines.

## supplementary crystallographic information

### Comment

Benzimidazole compounds and their metal complexes have been extensively investigated for their versatile properties such as biological activities (Küçükbay *et al.*, 2004; Çetinkaya *et al.*, 1996; Küçükbay *et al.*, 2009; Tavman *et al.*, 2005) and catalytic activities of their metal complexes in many organic syntheses (Küçükbay *et al.*,1996, Özdemir *et al.*, 2005). Contrary to an extensive chemistry about substituted alkyl derivatives of benzimidazole, there are a limited number of studies of alkylsilyl substituted benzimidazoles (Lukevics *et al.*, 2001). Alkylsilyl substituted benzimidazole derivatives exhibit important in vitro cytotoxicactivity. The insertion of the silicon atom into the *N*-alkyl chain in benzimidazoles increases the cytotoxic activity. For example,1-(3-trimethylsilylpropyl)benzimidazole inhibits carcinoma S-180 tumour growth in dose 1 mg kg^-1^ by 62 % (on ICR mice) (Lukevics *et al.*, 2001). The objective of the present study was to synthesize a trimethylsilylmethyl and NO_2 _substituted benzimidazole Co^II^ complex for the first time and compare it to those of related benzimidazole derivatives (Türktekin *et al.*, 2004; Pınar *et al.*, 2006; Akkurt *et al.*, 2005) reported previously.

In the title molecule (Fig. 1), the Co^II^ atom is coordinated in a distorted tetrahedral environment by two Cl atoms and two N atoms. The bond lengths involving the Co atoms are Co1—N1 = 2.013 (3), Co1—N4 = 2.013 (2), Co1—Cl1 = 2.2330 (10) and Co1—Cl2 = 2.2455 (9) Å, while the angles around the Co atom are Cl1— Co1— Cl2 = 112.94 (4), Cl1— Co1— N1 = 111.06 (8), Cl1— Co1— N4 = 110.18 (7), Cl2— Co1— N1 = 106.74 (7), Cl2— Co1— N4 = 111.81 (7) and N1— Co1— N4 = 103.67 (9)°. The average Co—N bond length of 2.013 (3)Å is almost equal to the value of 2.008 (2)Å in dichlorobis[1-(2-ethoxyethyl)-1*H*-benzimidazole-κ*N*^3^]cobalt(II) (Türktekin *et al.*, 2004) and 2.032 (2) Å inbis[1-(but-2-enyl)-5-nitro-1*H*-benzimidazole-*κN^3^*]\ dichlorocobalt(II) (Pınar *et al.*, 2006).

The Co—Cl bond lengths [2.2330 (10) and 2.2455 (9) Å] are comparable to the values of 2.2525 (8)Å inquinolinium trichloro(quinoline-*N*)cobaltate(II) (Pan & Xu, 2004) and 2.236 (1) Å in dichlorobis(1-propylimidazolidine-2-thione-κ*S*)cobalt(II) (Castro *et al.*, 2002) and 2.2680 (8) in bis[1-(but-2-enyl)-5-nitro-1*H*-benzimidazole-κ*N^3^*]\ dichlorocobalt(II)(Pınar *et al.*, 2006), but shorter than the value of 2.391 (1)Å observed in aquachlorobis(1,10-phenanthroline)cobalt(II)chloride dimethyl formamide solvate (Liu *et al.*, 2004).

The two benzimidazole ring systems N1/N2/C1–C7 and N4/N5/C12–C18 are almost planar, with maximum deviations of -0.037 (3) for C6 and -0.016 (2) for N4, respectively. The dihedral angle between them is 64.54 (10)°. The angles around the Si atoms with a distorted tetrahedral geometry rang from 106.02 (18)° to 113.60 (17)°.

The crystal structure of the title compound is stabilized by C—H···Cl and C—H···O hydrogen-bonding interactions (Fig. 2 and Table 1).

### Experimental

1-(Trimethylsilylmethyl)-5-nitrobenzimidazole used in this work as a starting compounds were prepared from reaction of 5(6)-methylbenzimidazole, (chloromethyl)trimethylsilane and KOH under reflux in EtOH.

A solution of 1-(trimethylsilylmethyl)-5-nitrobenzimidazole (2.0 g, 8.02 mmol) and cobalt(II) chloride (0.52 g, 4.01 mmol) in DMF (4 ml) was heated under reflux for 2 h. The mixture was then cooled to room temperature, after which the solvent was then removed from the filtrate in vacuo. The precipitate was then crystallized from EtOH / DMF(2:1). Yield : 2.30 g, 91%; m.p.: 473-474 K. Analysis calculated for C_22_H_30_N_6_O_4_Si_2_CoCl_2_.HCON(CH_3_)_2 _: C 42.80, H 5.32, N 13.97%. Found: C 42.79, H 5.31, N 13.92%. IR : ν_(C=N)_: 1523 cm^-1^. ^1^H-NMR (DMSO-d_6_): δ = 7.97 ppm (s, 2H, N=CH—N), 6.39 (m, 6H, Ar—H), 4.25 (m, 4H, CH_2_Si), -0.27 (s, 18H, Si (CH_3_)_3_).

### Refinement

All H atoms were placed at calculated positions and treated as riding atoms, with C—H = 0.95–0.99 Å, and U_iso_(H) = 1.2 or 1.5U_eq_(C).

### Crystal data

**Table:**

[CoCl_2_(C_11_H_15_N_3_O_2_Si)_2_]·C_3_H_7_NO	*Z* = 2
*M**_r_* = 701.63	*F*(000) = 730
Triclinic, *P*1	*D*_x_ = 1.428 Mg m^−^^3^
Hall symbol: -P 1	Mo *K*α radiation, λ = 0.71073 Å
*a* = 9.8982 (4) Å	Cell parameters from 4864 reflections
*b* = 11.6936 (5) Å	θ = 5.4–51.0°
*c* = 15.9293 (6) Å	µ = 0.81 mm^−^^1^
α = 106.041 (2)°	*T* = 100 K
β = 107.408 (2)°	Plate, blue
γ = 99.040 (3)°	0.20 × 0.12 × 0.08 mm
*V* = 1631.97 (12) Å^3^	

### Data collection

**Table:**

Brruker APEXII QUAZAR diffractometer	8851 independent reflections
Radiation source: ImuS	5342 reflections with *I* > 2σ(*I*)
multilayer	*R*_int_ = 0.059
ω and φ scans	θ_max_ = 29.4°, θ_min_ = 1.4°
Absorption correction: multi-scan (*SADABS*; Bruker, 2008)	*h* = −13→13
*T*_min_ = 0.890, *T*_max_ = 0.937	*k* = −16→16
30895 measured reflections	*l* = −21→21

### Refinement

**Table:**

Refinement on *F*^2^	Primary atom site location: structure-invariant direct methods
Least-squares matrix: full	Secondary atom site location: difference Fourier map
*R*[*F*^2^ > 2σ(*F*^2^)] = 0.053	Hydrogen site location: inferred from neighbouring sites
*wR*(*F*^2^) = 0.123	H-atom parameters constrained
*S* = 1.02	*w* = 1/[σ^2^(*F*_o_^2^) + (0.0506*P*)^2^ + 0.0752*P*] where *P* = (*F*_o_^2^ + 2*F*_c_^2^)/3
8851 reflections	(Δ/σ)_max_ = 0.001
387 parameters	Δρ_max_ = 0.90 e Å^−^^3^
0 restraints	Δρ_min_ = −0.48 e Å^−^^3^

### Special details

**Table:**

Experimental. Data were collected on an APEX II QUAZAR diffractometer equipped with a brilliant, high intense IµS (microfocus source) with multilayer mirrors for monochromation and collimation.
Geometry. Bond distances, angles etc. have been calculated using the rounded fractional coordinates. All su's are estimated from the variances of the (full) variance-covariance matrix. The cell esds are taken into account in the estimation of distances, angles and torsion angles
Refinement. Refinement on *F*^2^ for ALL reflections except those flagged by the user for potential systematic errors. Weighted *R*-factors wR and all goodnesses of fit *S* are based on *F*^2^, conventional *R*-factors *R* are based on *F*, with *F* set to zero for negative *F*^2^. The observed criterion of *F*^2^ > σ(*F*^2^) is used only for calculating -*R*-factor-obs etc. and is not relevant to the choice of reflections for refinement. *R*-factors based on *F*^2^ are statistically about twice as large as those based on *F*, and *R*-factors based on ALL data will be even larger.

### Fractional atomic coordinates and isotropic or equivalent isotropic displacement parameters (Å^2^)

**Table:**

	*x*	*y*	*z*	*U*_iso_*/*U*_eq_	
Co1	0.38557 (4)	0.69835 (4)	0.77352 (3)	0.0194 (1)	
Cl1	0.23442 (8)	0.51641 (7)	0.74255 (6)	0.0299 (2)	
Cl2	0.28764 (9)	0.80333 (7)	0.68299 (5)	0.0305 (3)	
Si1	0.80522 (9)	0.66034 (8)	0.52977 (6)	0.0220 (3)	
Si2	0.76085 (9)	0.79463 (8)	1.19186 (6)	0.0242 (3)	
O1	0.6900 (3)	0.3815 (2)	0.93714 (16)	0.0374 (8)	
O2	0.9030 (3)	0.3653 (2)	0.93302 (19)	0.0490 (10)	
O3	−0.0582 (2)	0.8517 (2)	0.90291 (16)	0.0357 (8)	
O4	−0.0395 (2)	0.8620 (2)	1.04422 (17)	0.0393 (8)	
N1	0.5723 (3)	0.6770 (2)	0.75277 (16)	0.0202 (8)	
N2	0.7576 (3)	0.7092 (2)	0.70128 (16)	0.0210 (8)	
N3	0.7937 (3)	0.4046 (2)	0.91233 (19)	0.0310 (9)	
N4	0.4540 (2)	0.7981 (2)	0.91071 (16)	0.0183 (7)	
N5	0.6010 (2)	0.8884 (2)	1.06061 (15)	0.0172 (7)	
N6	0.0135 (3)	0.8609 (2)	0.98339 (19)	0.0264 (8)	
C1	0.6695 (3)	0.6074 (3)	0.78068 (19)	0.0206 (9)	
C2	0.6672 (3)	0.5322 (3)	0.8338 (2)	0.0239 (9)	
C3	0.7870 (3)	0.4827 (3)	0.8532 (2)	0.0240 (10)	
C4	0.9025 (3)	0.5029 (3)	0.8219 (2)	0.0262 (10)	
C5	0.9033 (3)	0.5751 (3)	0.7674 (2)	0.0249 (10)	
C6	0.7850 (3)	0.6276 (3)	0.74777 (19)	0.0209 (9)	
C7	0.6315 (3)	0.7364 (3)	0.70664 (19)	0.0211 (9)	
C8	0.8477 (3)	0.7615 (3)	0.6555 (2)	0.0233 (9)	
C9	0.8366 (4)	0.5082 (3)	0.5292 (2)	0.0316 (11)	
C10	0.9278 (3)	0.7459 (3)	0.4868 (2)	0.0286 (10)	
C11	0.6093 (3)	0.6419 (3)	0.4624 (2)	0.0301 (11)	
C12	0.3698 (3)	0.8356 (3)	0.96419 (19)	0.0173 (9)	
C13	0.2206 (3)	0.8261 (3)	0.9372 (2)	0.0190 (9)	
C14	0.1713 (3)	0.8717 (3)	1.0084 (2)	0.0215 (9)	
C15	0.2602 (3)	0.9252 (3)	1.1030 (2)	0.0221 (9)	
C16	0.4089 (3)	0.9373 (3)	1.1298 (2)	0.0206 (9)	
C17	0.4614 (3)	0.8913 (3)	1.05910 (19)	0.0167 (8)	
C18	0.5901 (3)	0.8324 (3)	0.97189 (19)	0.0176 (8)	
C19	0.7347 (3)	0.9258 (3)	1.14482 (19)	0.0204 (9)	
C20	0.6391 (3)	0.7803 (3)	1.2593 (2)	0.0302 (11)	
C21	0.9569 (4)	0.8365 (4)	1.2669 (3)	0.0517 (16)	
C22	0.7128 (4)	0.6516 (3)	1.0898 (2)	0.0424 (14)	
O5	0.3510 (3)	1.0259 (2)	0.34832 (19)	0.0470 (10)	
N7	0.3118 (3)	0.8861 (2)	0.41855 (17)	0.0256 (8)	
C23	0.3349 (4)	0.9199 (4)	0.3519 (3)	0.0478 (16)	
C24	0.2906 (4)	0.9746 (4)	0.4965 (3)	0.0484 (14)	
C25	0.2834 (4)	0.7605 (3)	0.4171 (3)	0.0472 (14)	
H2	0.58920	0.51540	0.85550	0.0290*	
H4	0.98130	0.46640	0.83840	0.0310*	
H5	0.98000	0.58890	0.74420	0.0300*	
H7	0.58970	0.79200	0.68020	0.0250*	
H8A	0.95250	0.77410	0.69220	0.0280*	
H8B	0.83170	0.84310	0.65610	0.0280*	
H9A	0.81830	0.45610	0.46500	0.0470*	
H9B	0.76970	0.46860	0.55370	0.0470*	
H9C	0.93820	0.51920	0.56880	0.0470*	
H10A	1.03020	0.76020	0.52660	0.0430*	
H10B	0.90560	0.82520	0.48890	0.0430*	
H10C	0.91240	0.69750	0.42220	0.0430*	
H11A	0.59310	0.72290	0.46410	0.0450*	
H11B	0.54730	0.60370	0.49030	0.0450*	
H11C	0.58410	0.58930	0.39730	0.0450*	
H13	0.15650	0.79030	0.87350	0.0230*	
H15	0.21820	0.95320	1.14870	0.0270*	
H16	0.47250	0.97530	1.19340	0.0250*	
H18	0.67190	0.81890	0.95490	0.0210*	
H19A	0.72860	0.99600	1.19400	0.0250*	
H19B	0.82100	0.95400	1.12940	0.0250*	
H20A	0.65820	0.71800	1.28850	0.0450*	
H20B	0.53650	0.75540	1.21700	0.0450*	
H20C	0.65840	0.85980	1.30800	0.0450*	
H21A	0.97770	0.76980	1.29050	0.0780*	
H21B	0.97900	0.91240	1.31980	0.0780*	
H21C	1.01770	0.84960	1.23020	0.0780*	
H22A	0.77640	0.66230	1.05430	0.0640*	
H22B	0.61020	0.63470	1.04940	0.0640*	
H22C	0.72650	0.58240	1.11190	0.0640*	
H23	0.33990	0.85760	0.30080	0.0580*	
H24A	0.33100	1.05880	0.50050	0.0720*	
H24B	0.34120	0.96270	0.55530	0.0720*	
H24C	0.18560	0.96110	0.48560	0.0720*	
H25A	0.18070	0.73140	0.40970	0.0710*	
H25B	0.34760	0.75680	0.47610	0.0710*	
H25C	0.30270	0.70800	0.36470	0.0710*	

### Atomic displacement parameters (Å^2^)

**Table:**

	*U*^11^	*U*^22^	*U*^33^	*U*^12^	*U*^13^	*U*^23^
Co1	0.0190 (2)	0.0224 (2)	0.0177 (2)	0.0079 (2)	0.0067 (2)	0.0069 (2)
Cl1	0.0287 (4)	0.0262 (4)	0.0359 (4)	0.0061 (3)	0.0135 (4)	0.0110 (4)
Cl2	0.0374 (5)	0.0281 (4)	0.0214 (4)	0.0142 (4)	0.0023 (3)	0.0075 (3)
Si1	0.0215 (5)	0.0251 (5)	0.0226 (4)	0.0078 (4)	0.0107 (4)	0.0090 (4)
Si2	0.0231 (5)	0.0326 (5)	0.0214 (4)	0.0123 (4)	0.0080 (4)	0.0135 (4)
O1	0.0466 (15)	0.0345 (14)	0.0415 (14)	0.0148 (12)	0.0206 (12)	0.0211 (12)
O2	0.0418 (15)	0.0509 (17)	0.0700 (19)	0.0243 (13)	0.0154 (14)	0.0429 (15)
O3	0.0227 (12)	0.0408 (15)	0.0360 (14)	0.0129 (11)	0.0039 (11)	0.0066 (12)
O4	0.0273 (13)	0.0520 (16)	0.0509 (15)	0.0176 (12)	0.0260 (12)	0.0193 (13)
N1	0.0220 (13)	0.0228 (14)	0.0194 (13)	0.0090 (11)	0.0087 (11)	0.0094 (11)
N2	0.0212 (13)	0.0264 (14)	0.0209 (13)	0.0091 (11)	0.0118 (11)	0.0102 (11)
N3	0.0365 (17)	0.0254 (15)	0.0351 (16)	0.0097 (13)	0.0128 (14)	0.0157 (13)
N4	0.0162 (13)	0.0227 (13)	0.0195 (12)	0.0071 (11)	0.0082 (10)	0.0097 (11)
N5	0.0160 (12)	0.0194 (13)	0.0182 (12)	0.0076 (10)	0.0055 (10)	0.0086 (10)
N6	0.0215 (14)	0.0193 (14)	0.0369 (16)	0.0071 (11)	0.0116 (13)	0.0054 (12)
C1	0.0240 (16)	0.0192 (16)	0.0168 (15)	0.0085 (13)	0.0056 (13)	0.0040 (12)
C2	0.0283 (17)	0.0217 (16)	0.0220 (16)	0.0045 (14)	0.0102 (14)	0.0078 (13)
C3	0.0320 (18)	0.0171 (16)	0.0220 (16)	0.0087 (14)	0.0049 (14)	0.0094 (13)
C4	0.0234 (17)	0.0263 (17)	0.0272 (17)	0.0097 (14)	0.0057 (14)	0.0085 (14)
C5	0.0205 (16)	0.0278 (18)	0.0256 (16)	0.0079 (14)	0.0082 (13)	0.0071 (14)
C6	0.0200 (16)	0.0227 (16)	0.0199 (15)	0.0073 (13)	0.0060 (13)	0.0075 (13)
C7	0.0236 (16)	0.0269 (17)	0.0176 (15)	0.0119 (14)	0.0102 (13)	0.0089 (13)
C8	0.0241 (16)	0.0250 (17)	0.0259 (16)	0.0063 (14)	0.0148 (14)	0.0102 (14)
C9	0.043 (2)	0.0286 (19)	0.0313 (18)	0.0171 (16)	0.0195 (16)	0.0115 (15)
C10	0.0207 (16)	0.038 (2)	0.0291 (17)	0.0065 (15)	0.0111 (14)	0.0130 (15)
C11	0.0245 (17)	0.034 (2)	0.0329 (18)	0.0062 (15)	0.0098 (15)	0.0144 (16)
C12	0.0171 (15)	0.0176 (15)	0.0203 (15)	0.0058 (12)	0.0072 (12)	0.0099 (12)
C13	0.0186 (15)	0.0161 (15)	0.0221 (15)	0.0048 (12)	0.0034 (12)	0.0103 (12)
C14	0.0169 (15)	0.0194 (16)	0.0334 (17)	0.0078 (13)	0.0117 (13)	0.0125 (14)
C15	0.0254 (16)	0.0208 (16)	0.0259 (16)	0.0078 (13)	0.0153 (14)	0.0092 (13)
C16	0.0241 (16)	0.0216 (16)	0.0185 (15)	0.0088 (13)	0.0094 (13)	0.0072 (13)
C17	0.0174 (15)	0.0156 (15)	0.0200 (14)	0.0062 (12)	0.0072 (12)	0.0088 (12)
C18	0.0200 (15)	0.0193 (15)	0.0191 (14)	0.0084 (13)	0.0112 (13)	0.0085 (12)
C19	0.0175 (15)	0.0226 (16)	0.0192 (15)	0.0053 (13)	0.0053 (12)	0.0056 (13)
C20	0.0287 (18)	0.038 (2)	0.0277 (17)	0.0101 (16)	0.0114 (14)	0.0151 (16)
C21	0.026 (2)	0.088 (3)	0.058 (3)	0.020 (2)	0.0110 (19)	0.052 (3)
C22	0.074 (3)	0.036 (2)	0.038 (2)	0.030 (2)	0.032 (2)	0.0223 (18)
O5	0.0476 (16)	0.0460 (16)	0.0694 (19)	0.0174 (13)	0.0287 (14)	0.0420 (15)
N7	0.0230 (14)	0.0266 (15)	0.0286 (14)	0.0060 (12)	0.0061 (12)	0.0153 (12)
C23	0.039 (2)	0.062 (3)	0.055 (3)	0.018 (2)	0.020 (2)	0.033 (2)
C24	0.056 (3)	0.044 (2)	0.041 (2)	0.012 (2)	0.018 (2)	0.0085 (19)
C25	0.045 (2)	0.045 (2)	0.065 (3)	0.0190 (19)	0.021 (2)	0.034 (2)

### Geometric parameters (Å, °)

**Table:**

Co1—Cl1	2.2330 (10)	C14—C15	1.396 (4)
Co1—Cl2	2.2455 (9)	C15—C16	1.376 (4)
Co1—N1	2.013 (3)	C16—C17	1.390 (4)
Co1—N4	2.013 (2)	C2—H2	0.9500
Si1—C8	1.902 (3)	C4—H4	0.9500
Si1—C9	1.852 (4)	C5—H5	0.9500
Si1—C10	1.854 (3)	C7—H7	0.9500
Si1—C11	1.859 (3)	C8—H8B	0.9900
Si2—C19	1.904 (4)	C8—H8A	0.9900
Si2—C20	1.856 (3)	C9—H9A	0.9800
Si2—C21	1.851 (4)	C9—H9B	0.9800
Si2—C22	1.858 (3)	C9—H9C	0.9800
O1—N3	1.224 (4)	C10—H10A	0.9800
O2—N3	1.232 (4)	C10—H10B	0.9800
O3—N6	1.231 (4)	C10—H10C	0.9800
O4—N6	1.230 (4)	C11—H11B	0.9800
O5—C23	1.244 (5)	C11—H11C	0.9800
N1—C1	1.406 (4)	C11—H11A	0.9800
N1—C7	1.333 (4)	C13—H13	0.9500
N2—C7	1.356 (4)	C15—H15	0.9500
N2—C8	1.472 (4)	C16—H16	0.9500
N2—C6	1.373 (4)	C18—H18	0.9500
N3—C3	1.477 (4)	C19—H19B	0.9900
N4—C12	1.395 (4)	C19—H19A	0.9900
N4—C18	1.324 (4)	C20—H20A	0.9800
N5—C18	1.346 (4)	C20—H20B	0.9800
N5—C19	1.474 (4)	C20—H20C	0.9800
N5—C17	1.381 (4)	C21—H21A	0.9800
N6—C14	1.467 (4)	C21—H21B	0.9800
N7—C25	1.444 (5)	C21—H21C	0.9800
N7—C23	1.298 (5)	C22—H22B	0.9800
N7—C24	1.473 (5)	C22—H22C	0.9800
C1—C6	1.407 (4)	C22—H22A	0.9800
C1—C2	1.381 (5)	C23—H23	0.9500
C2—C3	1.390 (5)	C24—H24A	0.9800
C3—C4	1.390 (4)	C24—H24B	0.9800
C4—C5	1.369 (5)	C24—H24C	0.9800
C5—C6	1.400 (5)	C25—H25A	0.9800
C12—C13	1.386 (4)	C25—H25B	0.9800
C12—C17	1.408 (4)	C25—H25C	0.9800
C13—C14	1.374 (4)		
			
Co1···H2	3.4200	C22···O1	3.338 (4)
Co1···H13	3.2900	C22···C18	3.333 (5)
Cl1···N4	3.484 (3)	C23···C21^xi^	3.446 (6)
Cl1···C5^i^	3.564 (3)	C23···C24^viii^	3.557 (6)
Cl2···N1	3.420 (3)	C24···C23^viii^	3.557 (6)
Cl2···C7	3.546 (3)	C25···C9^iii^	3.597 (5)
Cl1···H5^i^	2.7900	C5···H13^v^	2.9300
Cl1···H22C^ii^	2.8200	C5···H8A	2.9400
Cl1···H10C^iii^	2.8600	C5···H9B	3.0600
Cl1···H20A^ii^	3.0600	C6···H9B	3.0800
Cl2···H7	3.0300	C8···H5	3.0100
Cl2···H10A^i^	2.8400	C9···H9C^xii^	3.0900
Cl2···H9A^iii^	3.0700	C9···H5	3.0900
Cl2···H16^iv^	2.9400	C13···H19B^iv^	3.0800
Cl2···H19A^iv^	2.6700	C16···H20B	3.0800
Si2···O4^v^	3.657 (2)	C16···H19A	2.9200
O1···C22	3.338 (4)	C18···H22B	2.9100
O1···C12^ii^	3.399 (4)	C19···H16	3.0200
O1···C17^ii^	3.329 (4)	C23···H24B^viii^	3.0000
O2···C14^ii^	3.208 (4)	C23···H21C^xi^	2.9900
O2···O4^ii^	3.223 (4)	C24···H20C^iv^	3.0200
O2···O2^vi^	3.178 (4)	C25···H11A	3.0600
O2···C15^ii^	3.335 (4)	C25···H9B^iii^	2.8400
O3···C5^i^	3.245 (4)	H2···O1	2.4500
O3···C1^i^	3.253 (4)	H2···Co1	3.4200
O3···O4^vii^	3.129 (3)	H4···O2	2.3800
O3···N2^i^	3.000 (3)	H5···H13^v^	2.5900
O3···C7^i^	3.413 (4)	H5···H9C	2.5700
O3···C6^i^	2.869 (4)	H5···Cl1^v^	2.7900
O3···N6^vii^	3.234 (4)	H5···C8	3.0100
O4···O3^vii^	3.129 (3)	H5···C9	3.0900
O4···Si2^i^	3.657 (2)	H5···H8A	2.5500
O4···O2^ii^	3.223 (4)	H7···Cl2	3.0300
O4···C19^i^	3.182 (4)	H7···H8B	2.5400
O5···C8^viii^	3.406 (4)	H7···O5^viii^	2.3200
O5···C7^viii^	3.132 (4)	H8A···C5	2.9400
O1···H2	2.4500	H8A···H5	2.5500
O1···H20B^ii^	2.6500	H8B···H7	2.5400
O2···H4	2.3800	H8B···O5^viii^	2.5400
O3···H13	2.4800	H9A···Cl2^iii^	3.0700
O4···H18^i^	2.6800	H9B···C5	3.0600
O4···H19B^i^	2.3800	H9B···C6	3.0800
O4···H15	2.4600	H9B···C25^iii^	2.8400
O4···H22A^i^	2.8000	H9B···H25B^iii^	2.5600
O4···H21C^i^	2.8900	H9C···H5	2.5700
O5···H8B^viii^	2.5400	H9C···C9^xii^	3.0900
O5···H7^viii^	2.3200	H10A···Cl2^v^	2.8400
O5···H24A	2.4200	H10C···Cl1^iii^	2.8600
O5···H15^ix^	2.8700	H11A···H24A^viii^	2.4000
N1···N4	3.166 (3)	H11A···C25	3.0600
N1···Cl2	3.420 (3)	H11A···H25B	2.5800
N1···N2	2.243 (4)	H11B···H11B^iii^	2.5900
N1···C18	3.397 (4)	H13···C5^i^	2.9300
N2···O3^v^	3.000 (3)	H13···Co1	3.2900
N2···N1	2.243 (4)	H13···O3	2.4800
N4···N5	2.231 (3)	H13···H5^i^	2.5900
N4···Cl1	3.484 (3)	H15···O5^xiii^	2.8700
N4···N1	3.166 (3)	H15···O4	2.4600
N5···N4	2.231 (3)	H16···Cl2^iv^	2.9400
N5···C12^iv^	3.337 (4)	H16···C19	3.0200
N6···O3^vii^	3.234 (4)	H16···H19A	2.5100
N6···N6^vii^	3.216 (4)	H18···O4^v^	2.6800
N5···H22B	2.9400	H19A···H16	2.5100
C1···O3^v^	3.253 (4)	H19A···C16	2.9200
C5···C9	3.485 (4)	H19A···Cl2^iv^	2.6700
C5···Cl1^v^	3.564 (3)	H19B···O4^v^	2.3800
C5···O3^v^	3.245 (4)	H19B···C13^iv^	3.0800
C6···C9	3.596 (4)	H20A···Cl1^ii^	3.0600
C6···O3^v^	2.869 (4)	H20B···C16	3.0800
C7···O5^viii^	3.132 (4)	H20B···O1^ii^	2.6500
C7···O3^v^	3.413 (4)	H20C···C24^iv^	3.0200
C8···O5^viii^	3.406 (4)	H20C···H24B^iv^	2.5600
C9···C6	3.596 (4)	H21A···H25A^x^	2.5100
C9···C25^iii^	3.597 (5)	H21C···O4^v^	2.8900
C9···C5	3.485 (4)	H21C···C23^x^	2.9900
C12···O1^ii^	3.399 (4)	H22A···O4^v^	2.8000
C12···N5^iv^	3.337 (4)	H22B···N5	2.9400
C12···C17^iv^	3.532 (5)	H22B···C18	2.9100
C13···C19^iv^	3.515 (5)	H22C···Cl1^ii^	2.8200
C14···O2^ii^	3.208 (4)	H23···H25C	2.2900
C15···O2^ii^	3.335 (4)	H24A···O5	2.4200
C16···C18^iv^	3.506 (5)	H24A···H11A^viii^	2.4000
C17···C18^iv^	3.492 (5)	H24B···H25B	2.4100
C17···O1^ii^	3.329 (4)	H24B···C23^viii^	3.0000
C17···C12^iv^	3.532 (5)	H24B···H20C^iv^	2.5600
C18···C22	3.333 (5)	H25A···H21A^xi^	2.5100
C18···C16^iv^	3.506 (5)	H25B···H11A	2.5800
C18···C17^iv^	3.492 (5)	H25B···H24B	2.4100
C19···C13^iv^	3.515 (5)	H25B···H9B^iii^	2.5600
C19···O4^v^	3.182 (4)	H25C···H23	2.2900
C21···C23^x^	3.446 (6)		
			
Cl1—Co1—Cl2	112.94 (4)	C4—C5—H5	122.00
Cl1—Co1—N1	111.06 (8)	N1—C7—H7	124.00
Cl1—Co1—N4	110.18 (7)	N2—C7—H7	123.00
Cl2—Co1—N1	106.74 (7)	Si1—C8—H8A	109.00
Cl2—Co1—N4	111.81 (7)	H8A—C8—H8B	108.00
N1—Co1—N4	103.67 (9)	Si1—C8—H8B	109.00
C8—Si1—C9	108.71 (15)	N2—C8—H8A	109.00
C8—Si1—C10	105.46 (15)	N2—C8—H8B	109.00
C8—Si1—C11	107.76 (14)	Si1—C9—H9A	110.00
C9—Si1—C10	113.60 (17)	Si1—C9—H9B	109.00
C9—Si1—C11	109.95 (17)	Si1—C9—H9C	110.00
C10—Si1—C11	111.09 (15)	H9B—C9—H9C	109.00
C19—Si2—C20	108.88 (15)	H9A—C9—H9B	109.00
C19—Si2—C21	106.02 (18)	H9A—C9—H9C	110.00
C19—Si2—C22	107.56 (14)	Si1—C10—H10B	109.00
C20—Si2—C21	111.80 (17)	H10A—C10—H10C	109.00
C20—Si2—C22	110.98 (17)	Si1—C10—H10C	109.00
C21—Si2—C22	111.36 (19)	H10A—C10—H10B	110.00
Co1—N1—C1	132.6 (2)	Si1—C10—H10A	109.00
Co1—N1—C7	122.7 (2)	H10B—C10—H10C	109.00
C1—N1—C7	104.7 (3)	Si1—C11—H11C	109.00
C6—N2—C7	107.3 (3)	H11A—C11—H11C	110.00
C6—N2—C8	127.5 (3)	H11B—C11—H11C	109.00
C7—N2—C8	125.2 (3)	H11A—C11—H11B	109.00
O1—N3—O2	123.7 (3)	Si1—C11—H11A	109.00
O1—N3—C3	118.3 (3)	Si1—C11—H11B	109.00
O2—N3—C3	118.0 (3)	C14—C13—H13	122.00
Co1—N4—C12	128.49 (18)	C12—C13—H13	122.00
Co1—N4—C18	126.50 (19)	C14—C15—H15	120.00
C12—N4—C18	104.7 (2)	C16—C15—H15	120.00
C17—N5—C18	107.4 (2)	C17—C16—H16	122.00
C17—N5—C19	126.3 (2)	C15—C16—H16	122.00
C18—N5—C19	125.9 (2)	N4—C18—H18	123.00
O3—N6—O4	123.7 (3)	N5—C18—H18	123.00
O3—N6—C14	118.2 (3)	Si2—C19—H19A	109.00
O4—N6—C14	118.1 (3)	H19A—C19—H19B	108.00
C24—N7—C25	114.4 (3)	Si2—C19—H19B	109.00
C23—N7—C24	120.3 (3)	N5—C19—H19A	109.00
C23—N7—C25	124.7 (3)	N5—C19—H19B	109.00
N1—C1—C6	108.8 (3)	Si2—C20—H20A	109.00
N1—C1—C2	130.4 (3)	Si2—C20—H20B	109.00
C2—C1—C6	120.8 (3)	Si2—C20—H20C	109.00
C1—C2—C3	115.2 (3)	H20B—C20—H20C	110.00
N3—C3—C2	118.0 (3)	H20A—C20—H20B	109.00
N3—C3—C4	117.5 (3)	H20A—C20—H20C	109.00
C2—C3—C4	124.6 (3)	Si2—C21—H21B	109.00
C3—C4—C5	120.3 (3)	H21A—C21—H21C	110.00
C4—C5—C6	116.4 (3)	Si2—C21—H21C	109.00
N2—C6—C1	106.2 (3)	H21A—C21—H21B	109.00
N2—C6—C5	131.0 (3)	Si2—C21—H21A	109.00
C1—C6—C5	122.7 (3)	H21B—C21—H21C	109.00
N1—C7—N2	113.0 (3)	Si2—C22—H22C	109.00
Si1—C8—N2	113.4 (2)	H22A—C22—H22C	109.00
C13—C12—C17	120.4 (3)	H22B—C22—H22C	110.00
N4—C12—C17	109.3 (3)	H22A—C22—H22B	109.00
N4—C12—C13	130.3 (3)	Si2—C22—H22A	109.00
C12—C13—C14	115.7 (3)	Si2—C22—H22B	109.00
N6—C14—C13	117.6 (3)	O5—C23—N7	126.4 (4)
N6—C14—C15	117.7 (3)	O5—C23—H23	117.00
C13—C14—C15	124.7 (3)	N7—C23—H23	117.00
C14—C15—C16	119.8 (3)	N7—C24—H24A	109.00
C15—C16—C17	116.7 (3)	N7—C24—H24B	109.00
N5—C17—C12	105.2 (2)	N7—C24—H24C	109.00
N5—C17—C16	132.0 (3)	H24A—C24—H24B	109.00
C12—C17—C16	122.8 (3)	H24A—C24—H24C	110.00
N4—C18—N5	113.3 (3)	H24B—C24—H24C	109.00
Si2—C19—N5	112.1 (2)	N7—C25—H25A	109.00
C1—C2—H2	122.00	N7—C25—H25B	109.00
C3—C2—H2	122.00	N7—C25—H25C	110.00
C3—C4—H4	120.00	H25A—C25—H25B	109.00
C5—C4—H4	120.00	H25A—C25—H25C	109.00
C6—C5—H5	122.00	H25B—C25—H25C	109.00
			
Cl1—Co1—N1—C1	−47.5 (3)	Co1—N4—C18—N5	−174.0 (2)
Cl2—Co1—N1—C1	−171.0 (2)	C17—N5—C19—Si2	86.6 (3)
N4—Co1—N1—C1	70.8 (3)	C18—N5—C17—C12	−1.0 (4)
Cl1—Co1—N1—C7	134.4 (2)	C19—N5—C18—N4	173.7 (3)
Cl2—Co1—N1—C7	10.9 (2)	C19—N5—C17—C12	−174.2 (3)
N4—Co1—N1—C7	−107.3 (2)	C17—N5—C18—N4	0.4 (4)
Cl1—Co1—N4—C12	−58.2 (3)	C18—N5—C19—Si2	−85.4 (3)
Cl2—Co1—N4—C12	68.3 (3)	C19—N5—C17—C16	6.6 (6)
N1—Co1—N4—C12	−177.1 (3)	C18—N5—C17—C16	179.8 (4)
Cl1—Co1—N4—C18	114.8 (3)	O3—N6—C14—C15	157.4 (3)
Cl2—Co1—N4—C18	−118.7 (3)	O4—N6—C14—C15	−22.3 (4)
N1—Co1—N4—C18	−4.1 (3)	O3—N6—C14—C13	−23.9 (4)
C11—Si1—C8—N2	60.1 (3)	O4—N6—C14—C13	156.4 (3)
C10—Si1—C8—N2	178.8 (2)	C24—N7—C23—O5	4.9 (6)
C9—Si1—C8—N2	−59.0 (3)	C25—N7—C23—O5	175.4 (4)
C22—Si2—C19—N5	41.2 (3)	C2—C1—C6—C5	−0.8 (5)
C20—Si2—C19—N5	−79.2 (2)	N1—C1—C6—N2	0.5 (3)
C21—Si2—C19—N5	160.4 (2)	C6—C1—C2—C3	1.6 (4)
C7—N1—C1—C6	−0.9 (3)	C2—C1—C6—N2	−177.7 (3)
Co1—N1—C1—C6	−179.3 (2)	N1—C1—C6—C5	177.3 (3)
C1—N1—C7—N2	1.0 (3)	N1—C1—C2—C3	−176.1 (3)
C7—N1—C1—C2	177.0 (3)	C1—C2—C3—C4	−1.0 (5)
Co1—N1—C7—N2	179.59 (18)	C1—C2—C3—N3	178.1 (3)
Co1—N1—C1—C2	−1.4 (5)	C2—C3—C4—C5	−0.5 (5)
C7—N2—C6—C1	0.2 (3)	N3—C3—C4—C5	−179.6 (3)
C8—N2—C7—N1	−179.4 (3)	C3—C4—C5—C6	1.3 (5)
C6—N2—C7—N1	−0.8 (3)	C4—C5—C6—C1	−0.7 (5)
C7—N2—C8—Si1	−95.2 (3)	C4—C5—C6—N2	175.3 (3)
C8—N2—C6—C5	2.3 (5)	C17—C12—C13—C14	−1.2 (5)
C8—N2—C6—C1	178.8 (3)	C13—C12—C17—C16	1.0 (6)
C7—N2—C6—C5	−176.4 (3)	N4—C12—C17—N5	1.2 (4)
C6—N2—C8—Si1	86.4 (3)	N4—C12—C17—C16	−179.5 (3)
O1—N3—C3—C2	4.0 (4)	N4—C12—C13—C14	179.4 (3)
O2—N3—C3—C2	−176.8 (3)	C13—C12—C17—N5	−178.4 (3)
O1—N3—C3—C4	−176.9 (3)	C12—C13—C14—N6	−178.5 (3)
O2—N3—C3—C4	2.3 (4)	C12—C13—C14—C15	0.0 (6)
C12—N4—C18—N5	0.3 (4)	N6—C14—C15—C16	−180.0 (3)
C18—N4—C12—C17	−1.0 (4)	C13—C14—C15—C16	1.4 (6)
Co1—N4—C12—C17	173.2 (2)	C14—C15—C16—C17	−1.6 (5)
Co1—N4—C12—C13	−7.3 (5)	C15—C16—C17—N5	179.6 (4)
C18—N4—C12—C13	178.6 (4)	C15—C16—C17—C12	0.5 (5)

Symmetry codes: (i) *x*−1, *y*, *z*; (ii) −*x*+1, −*y*+1, −*z*+2; (iii) −*x*+1, −*y*+1, −*z*+1; (iv) −*x*+1, −*y*+2, −*z*+2; (v) *x*+1, *y*, *z*; (vi) −*x*+2, −*y*+1, −*z*+2; (vii) −*x*, −*y*+2, −*z*+2; (viii) −*x*+1, −*y*+2, −*z*+1; (ix) *x*, *y*, *z*−1; (x) *x*+1, *y*, *z*+1; (xi) *x*−1, *y*, *z*−1; (xii) −*x*+2, −*y*+1, −*z*+1; (xiii) *x*, *y*, *z*+1.

### Hydrogen-bond geometry (Å, °)

**Table:**

*D*—H···*A*	*D*—H	H···*A*	*D*···*A*	*D*—H···*A*
C5—H5···Cl1^v^	0.95	2.79	3.564 (3)	140
C7—H7···O5^viii^	0.95	2.32	3.132 (4)	143
C8—H8B···O5^viii^	0.99	2.54	3.406 (4)	145
C19—H19A···Cl2^iv^	0.99	2.67	3.659 (3)	175
C19—H19B···O4^v^	0.99	2.38	3.182 (4)	137
C22—H22C···Cl1^ii^	0.98	2.82	3.695 (3)	149
C24—H24A···O5	0.98	2.42	2.793 (5)	102

Symmetry codes: (v) *x*+1, *y*, *z*; (viii) −*x*+1, −*y*+2, −*z*+1; (iv) −*x*+1, −*y*+2, −*z*+2; (ii) −*x*+1, −*y*+1, −*z*+2.
